# Pandemic *Vibrio parahaemolyticus*, Maryland, USA, 2012

**DOI:** 10.3201/eid2004.130818

**Published:** 2014-04

**Authors:** Julie Haendiges, Marvin Rock, Robert A. Myers, Eric W. Brown, Peter Evans, Narjol Gonzalez-Escalona

**Affiliations:** Department of Health and Mental Hygiene, Baltimore, Maryland, USA (J. Haendiges, M. Rock, R.A. Myers);; Food and Drug Administration, College Park, Maryland, USA (E.W. Brown, P. Evans, N. Gonzalez-Escalona)

**Keywords:** bacteria, pandemic, Vibrio parahaemolyticus, Maryland, USA, United States, cross contamination, food preparation, traceback, PFGE pattern, pulsed-field gel electrophoresis pattern, seafood, epidemiology, gastroenteritis

**To the** **Editor:** Since 1996, an increasing number of infections caused by *Vibrio parahaemolyticus* strains belonging to a pandemic clonal complex (CC), CC3, typically O3:K6, have been observed worldwide (*1*–*3*); most of these strains are sequence type (ST) 3. In the summer of 1998, outbreaks linked to O3:K6 occurred in Galveston Bay, Texas, and Oyster Bay, New York, USA; the illnesses were associated with oyster consumption (*4*). Strains belonging to CC36 are the leading cause of *V. parahaemolyticus* infections in the United States. These strains are endemic to the West Coast (*2*) and have been historically linked to outbreak-associated *V. parahaemolyticus* infections caused by consumption of raw oysters harvested from the region (*5*).

In August 2012, a *V*. *parahaemolyticus* outbreak involving 6 persons occurred in Maryland, USA. The patients (members of 2 dining parties) had eaten in the same restaurant on the same day; raw and cooked seafood was served at the restaurant. Party A comprised 4 diners, of whom 2 had laboratory-confirmed illness and 2 were probable case-patients. Party B comprised 2 diners, of whom 1 had laboratory-confirmed illness and 1 was a probable case-patient. Probable case-patients were epidemiologically linked to confirmed case-patients, but *V. parahaemolyticus* was not detected in their stool samples. The epidemiologic investigation did not conclusively identify the specific food responsible for the outbreak. The affected diners had not eaten oysters, lobster, or mussels, but they had eaten cooked clams, fish, crab, and shrimp. Because the patients had not eaten oysters, a traceback investigation was not conducted. The outbreak possibly was caused by cross-contamination during food preparation. No other cases were reported from this restaurant or the surrounding area.

*V*. *parahaemolyticus* was isolated from stool samples of 3 of the patients. The isolates were characterized by real-time PCR for virulence-related genes (*tdh* and *trh*). All 3 isolates were *tdh* positive and lacked the *trh* gene. Pulsed-field gel electrophoresis (PFGE) was run, using *Sfil* and *Notl*; the resulting K16S12.0138 (*Sfil*) and K16N11.0143 (*Notl*) patterns were indistinguishable. The PFGE pattern combination was queried against combination entries made in PulseNet (www.cdc.gov/pulsenet/) during February 4, 2010–April 16, 2013, and found to be indistinguishable from other clinical entries (Technical Appendix 1, Table). This PFGE pattern combination has been seen 25 times; all patterns were for strains from humans (N. Facundo, pers. comm.). In 2012, this PFGE pattern combination was observed in 3 US states—California (6 cases), Arizona (6 cases), and Texas (5 cases)—but those isolates were not further tested (S.G. Stroika, pers. comm.), suggesting that other cases of pandemic *V. parahaemolyticus* infections have occurred in the United States but were not identified as being caused by pandemic clones.

The whole genomes of the 3 Maryland strains were sequenced by using the Ion Torrent personal genome machine (Life Technologies, Grand Island, NY, USA); in silico multilocus sequence typing (MLST) (*2*) showed that the isolates were all ST3, the most common ST belonging to CC3. Bioinformatic analysis of the whole genomes was conducted with the Bacterial Isolate Genome Sequence Database (*6*) genome comparator tool available within the *V*. *parahaemolyticus* MLST database (http://pubmlst.org/vparahaemolyticus) (*7*,*8*). Results confirmed that these outbreak isolates were linked to the O3:K6 pandemic clone of *V*. *parahaemolyticus* (Figure). We identified 2,613 variable loci in this analysis by using as reference genome the prototype pandemic *V. parahaemolyticus* clonal strain RIMD221633 (available from GenBank, www.ncbi.nlm.nih.gov/genome/?term=vibrioparahaemolyticus) (*10*). Differences in variable loci and the absence of certain genes indicated that, although indistinguishable by MLST and PFGE, these strains are easily differentiated from RIMD2210633 (Technical Appendix 1). The draft genome sequences for the 3 strains are available at the *V*. *parahaemolyticus* MLST database (identification nos. 1187 [Vp16MD], 1188 [Vp17MD], and 1189 [Vp18MD]).

**Figure:**
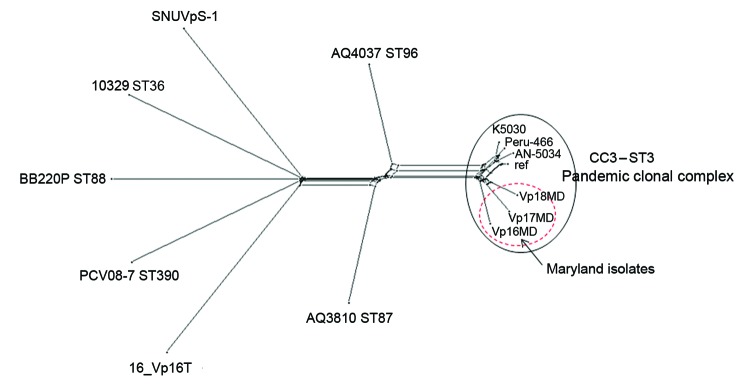
Neighbor-Net graph generated with the Bacterial Isolate Genome Sequence Database genome (BIGSdb) comparator tool implemented within the *Vibrio* *parahaemolyticus* MLST database (http://pubmlst.org/vparahaemolyticus) (*7*,*8*) using 2,613 variable loci. These loci were identified by using as a reference (ref) the *V. parahaemolyticus* strain RIMD2210633 chromosome I (3,080 genes) and conducting a whole-genome MLST (wgMLST) for *V. parahaemolyticus* genomes available through GenBank (AN-5034 O4:K68 ST3, Peru-466 ST3, K5030 ST3, 16_VP16T, AQ3810 ST87, AQ4037 ST96, PCV08–7 ST390, BB220P ST88, and SNUVpS-1) and 3 Maryland outbreak strains (Vp16MD, Vp17MD, and Vp18MD). This typing showed that these 3 strains belonged to the pandemic CC3. A similar graph was obtained by using chromosome II of the same strain as reference (data not shown). In brief, the BIGSdb genome comparator tool performs wgMLST, which produces a color-coded wgMLST output (Technical Appendix 2) that facilitates comparison among isolates. This loci output is further categorized into loci that are 1) variable among all isolates, 2) identical among all isolates, 3) missing in all isolates, and 4) incomplete because of being located at the ends of contigs. The variable loci among all isolates are the loci used for assessing relationships and producing a distance matrix based on the number of variable alleles; the strains are resolved into a network by using the NeighborNet algorithm (*9*). MLST, multilocus sequence typing; CC, clonal complex; ST, sequence type.

*V. parahaemolyticus* strains belonging to the pandemic CC have caused thousands of infections and a *V*. *parahaemolyticus* pandemic (*3*). Foodborne illnesses caused by pandemic *V. parahaemolyticus* are uncommonly reported in the United States. In Maryland, 12 and 21 cases of *V. parahaemolyticus–*associated gastroenteritis were reported in 2012 and 2013, respectively. We report that the pandemic CC was still causing US outbreaks as recently as August 2012. It is possible that complete availability of PFGE patterns during the outbreaks (online Technical Appendix 1) could have provided additional insight into the scope of the outbreak and implicated food sources. The application of rapid, whole-genome sequencing technology aided our discovery that the Maryland outbreak strains were part of the pandemic CC and likely related to *V. parahaemolyticus* strains that shared common PFGE patterns and that were reported as the cause of illnesses in several states around the same time as the Maryland outbreak.

The presence of this virulent *V. parahaemolyticus* strain in Maryland is an ongoing public health concern, requiring continued microbiological surveillance. This pandemic strain also indicates the need for establishing a *V. parahaemolyticus* genome database that is accessible worldwide. Such a database would enable improved tracking and faster responses to emergent and dangerous pandemic clonal strains.

Technical Appendix 1 Isolates uploaded into PulseNet during February 4, 2010–April 16, 2013, that matched the pulsed-field gel electrophoresis pattern combination, K16S12.0138 and K16N11.0143, of isolates identified during an outbreak of *Vibrio parahaemolyticus* in Maryland, USA, August 2012.

Technical Appendix 2 Loci differences and presence or absence in the 3 *Vibrio parahaemolyticus* outbreak strains (Vp16MD, Vp17MD, and Vp18MD) from Maryland, USA, compared with chromosome I and II of RIMD221633, the prototypic *V. parahaemolyticus* pandemic strain.

## References

[1] González-Escalona N, Cachicas V, Acevedo C, Rioseco ML, Vergara JA, Cabello F, *Vibrio parahaemolyticus* diarrhea, Chile, 1998 and 2004. Emerg Infect Dis. 2005;11:129–31. 10.3201/eid1101.040762 15705337 PMC3294363

[2] González-Escalona N, Martinez-Urtaza J, Romero J, Espejo RT, Jaykus LA, DePaola A. Determination of molecular phylogenetics of *Vibrio parahaemolyticus* strains by multilocus sequence typing. J Bacteriol. 2008;190:2831–40. 10.1128/JB.01808-07 18281404 PMC2293261

[3] Nair GB, Ramamurthy T, Bhattacharya SK, Dutta B, Takeda Y, Sack DA. Global dissemination of *Vibrio parahaemolyticus* serotype O3:K6 and its serovariants. Clin Microbiol Rev. 2007;20:39–48. 10.1128/CMR.00025-06 17223622 PMC1797631

[4] DePaola A, Kaysner CA, Bowers J, Cook DW. Environmental investigations of *Vibrio parahaemolyticus* in oysters after outbreaks in Washington, Texas, and New York (1997 and 1998). Appl Environ Microbiol. 2000;66:4649–54. 10.1128/AEM.66.11.4649-4654.2000 11055906 PMC92362

[5] Abbott SL, Powers C, Kaysner CA, Takeda Y, Ishibashi M, Joseph SW, Emergence of a restricted bioserovar of *Vibrio parahaemolyticus* as the predominant cause of vibrio-associated gastroenteritis on the West Coast of the United States and Mexico. J Clin Microbiol. 1989;27:2891–3 . 2592553 10.1128/jcm.27.12.2891-2893.1989 PMC267157

[6] Jolley KA, Maiden MC. BIGSdb: scalable analysis of bacterial genome variation at the population level. BMC Bioinformatics. 2010;11:595. 10.1186/1471-2105-11-595 21143983 PMC3004885

[7] Jolley KA, Hill DM, Bratcher HB, Harrison OB, Feavers IM, Parkhill J, Resolution of a meningococcal disease outbreak from whole-genome sequence data with rapid Web-based analysis methods. J Clin Microbiol. 2012;50:3046–53. 10.1128/JCM.01312-12 22785191 PMC3421817

[8] Jolley KA, Maiden MC. Automated extraction of typing information for bacterial pathogens from whole genome sequence data: *Neisseria meningitidis* as an exemplar. Euro Surveill. 2013;18:20379 . 23369391 10.2807/ese.18.04.20379-en PMC3977036

[9] Bryant D, Moulton V. Neighbor-net: an agglomerative method for the construction of phylogenetic networks. Mol Biol Evol. 2004;21:255–65. 10.1093/molbev/msh018 14660700

[10] Makino K, Oshima K, Kurokawa K, Yokoyama K, Uda T, Tagomori K, Genome sequence of *Vibrio parahaemolyticus*: a pathogenic mechanism distinct from that of *V. cholerae.* Lancet. 2003;361:743–9. 10.1016/S0140-6736(03)12659-1 12620739

