# Supplementary material for: Pandemic Vibrio parahaemolyticus, Maryland, USA, 2012
Source: Emerg Infect Dis. 2014 Apr;20(4):718–20. doi: 10.3201/eid2004.130818 (PMC3966373; doi:10.3201/eid2004.130818)
Supplement: Technical Appendix 1 — Isolates uploaded into PulseNet during February 4, 2010–April 16, 2013, that matched the pulsed-field gel electrophoresis pattern combination, K16S12.0138 and K16N11.0143, of isolates identified during an outbreak of Vibrio parahaemolyticus in Maryland, USA, August 2012. [file 13-0818-Techapp-s1.pdf]

Article DOI: http://dx.doi.org/10.3201/eid2004.130818

# Pandemic *Vibrio parahaemolyticus*, Maryland, USA, 2012

## Technical Appendix

Table. Isolates that matched PFGE patterns K16S12.0138 and K16N11.0143 combination against PulseNet entries uploaded during February 4, 2010–April 16, 2013 and associated data*

| Isolate | Source state | Source type | Patient age, y | Patient sex | Isolate date | Upload date | PFGE-SfiI-pattern | PFGE-NotI-pattern |
|---|---|---|---|---|---|---|---|---|
| TX___TXACB1000107 | TX | Human | 42 | M | 1/15/2010 | 2/4/2010 | K16S12.0138 | K16N11.0143 |
| CDC__AM47597 | WA | Human | | M | 6/11/2011 | 10/19/2011 | K16S12.0138 | K16N11.0143 |
| CDC__AM47598 | WA | Human | | M | 6/14/2011 | 10/19/2011 | K16S12.0138 | K16N11.0143 |
| CDC__101215235 | NY | Human | 33 | M | 7/9/2011 | 8/2/2012 | K16S12.0138 | K16N11.0143 |
| CDC__PI11200004 H | AZ | Human | 38 | F | 7/11/2011 | 10/15/2012 | K16S12.0138 | K16N11.0143 |
| CDC__PI11200004 NH | AZ | Human | 38 | F | 7/11/2011 | 10/15/2012 | K16S12.0138 | K16N11.0143 |
| CDC__AM47605 | WA | Human | | M | 7/20/2011 | 10/19/2011 | K16S12.0138 | K16N11.0143 |
| TX___TXACB1102640 | TX | Human | 56 | M | 8/8/2011 | 9/8/2011 | K16S12.0138 | K16N11.0143 |
| CDC__AM47611 | WA | Human | | M | 8/18/2011 | 11/2/2011 | K16S12.0138 | K16N11.0143 |
| CDC__AM47614 | WA | Human | | M | 8/30/2011 | 11/2/2011 | K16S12.0138 | K16N11.0143 |
| CDC__AM47617 | WA | Human | | M | 9/23/2011 | 11/2/2011 | K16S12.0138 | K16N11.0143 |
| CDC__PI11272013 | AZ | Human | 27 | M | 9/25/2011 | 8/2/2012 | K16S12.0138 | K16N11.0143 |
| CA___M12X02339 | CA | Human | 39 | M | 6/15/2012 | 7/23/2012 | K16S12.0138 | K16N11.0143 |
| CA___M12X02915 | CA | Human | 39 | F | 7/6/2012 | 7/27/2012 | K16S12.0138 | K16N11.0143 |
| MD___MDA12147539 | MD | Human | 53 | M | 8/21/2012 | 10/9/2012 | K16S12.0138 | K16N11.0143 |
| MD___MDA12148581 | MD | Human | 49 | F | 8/22/2012 | 10/9/2012 | K16S12.0138 | K16N11.0143 |
| MD___MDA12162046 | MD | Human | 51 | NK | 8/24/2012 | 10/9/2012 | K16S12.0138 | K16N11.0143 |
| TX___TXACB1202640 | TX | Human | 67 | F | 8/20/2012 | 10/10/2012 | K16S12.0138 | K16N11.0143 |
| CDC__PI12255002 | AZ | Human | | NK | | 11/5/2012 | K16S12.0138 | K16N11.0143 |
| CDC__PI12256006 | AZ | Human | | NK | | 11/5/2012 | K16S12.0138 | K16N11.0143 |
| CDC__M12X4985 G | CA | Human | | | | 1/28/2013 | K16S12.0138 | K16N11.0143 |
| CDC__M12X4985 W | CA | Human | | | | 1/28/2013 | K16S12.0138 | K16N11.0143 |
| CDC__M12X05012 G | CA | Human | | | | 1/28/2013 | K16S12.0138 | K16N11.0143 |
| CDC__M12X05012 W | CA | Human | | | | 1/28/2013 | K16S12.0138 | K16N11.0143 |
| NY___IDR1300009145 | NY | Human | 49 | M | 3/29/2013 | 4/16/2013 | K16S12.0138 | K16N11.0143 |

* PFGE, pulsed-field gel electrophoresis; NK, not known.
